# Chitosan Oleate Coated Poly Lactic-Glycolic Acid (PLGA) Nanoparticles versus Chitosan Oleate Self-Assembled Polymeric Micelles, Loaded with Resveratrol

**DOI:** 10.3390/md17090515

**Published:** 2019-09-01

**Authors:** Dalila Miele, Laura Catenacci, Milena Sorrenti, Silvia Rossi, Giuseppina Sandri, Lorenzo Malavasi, Giacomo Dacarro, Franca Ferrari, Maria Cristina Bonferoni

**Affiliations:** 1 Department of Drug Sciences, University of Pavia, Viale Taramelli 12, 27100 Pavia, Italy; 2 Department of Chemistry, University of Pavia, Viale Taramelli 14, 27100 Pavia, Italy

**Keywords:** chitosan oleate salt, polymeric micelles, PLGA, nanoparticles, resveratrol

## Abstract

Chitosan oleate (CS-OA), a chitosan salt with amphiphilic properties, has demonstrated the ability to self-assemble in aqueous environment to give polymeric micelles useful to load poorly soluble drugs. More recently, CS-OA was proposed to stabilize nanoemulsions during the preparation by emulsification and solvent evaporation of poly lactic-glycolic acid (PLGA) nanoparticles (NPs) loaded with curcumin. Positive mucoadhesive behavior and internalization properties were demonstrated for these NPs attributable to the presence of positive charge at the NP surface. In the present paper, two CS-OA-based nanosystems, micelles and PLGA NPs, were compared with the aim of elucidating their physico-chemical characteristics, and especially their interaction with cell substrates. The two systems were loaded with resveratrol (RSV), a hydrophobic polyphenol endowed with anti-cancerogenic, anti-inflammatory, and heart/brain protective effects, but with low bioavailability mainly due to poor aqueous solubility. Calorimetric analysis and X-ray spectra demonstrated amorphization of RSV, confirming its affinity for hydrophobic domains of polymeric micelles and PLGA core of NPs. TGA decomposition patterns suggest higher stability of PLGA-NPs compared with polymeric micelles, that anyway resulted more stable than expected, considering the RSV release profiles, and the cell line interaction results.

## 1. Introduction

Many reports have been focused on the preparation and on the possible exploitation of amphiphilic derivatives of chitosan [1]. Chitosan (CS) and chitosan derivatives have been hydrophobically modified by interaction with different hydrophobic pendant groups, usually by covalent derivatization [2,3,4,5,6]. Less common in the literature is the design and characterization of amphiphilic derivatives based on ionic interaction between the polymer and the hydrophobic moieties [7,8].

Amphiphilic polymers are known for their ability to self-assemble into nanoparticles with a hydrophobic core or inner hydrophobic domains and outer shell layers made by the chains of the polymer hydrophilic portions. This mechanism, common to polymeric micelles, was also previously shown for chitosan oleate (CS-OA), a chitosan salt with amphiphilic properties and whose self-assembling in aqueous environment resulted suitable to efficiently load hydrophobic poorly soluble molecules [7,9].

In the case of chitosan amphiphilic derivatives, this behavior results in nanocarriers that maintain the favorable biological properties of the polysaccharide, among which are mucoadhesion, penetration enhancement, or antibacterial effect [1,6,10,11].

More recently, CS-OA was proposed to stabilize nanoemulsions during the preparation by emulsification and solvent evaporation of poly lactic-glycolic acid (PLGA) nanoparticles (NPs). It was demonstrated that the resulting NPs were suitably loaded with curcumin in the PLGA hydrophobic core and maintained a chitosan shell on the surface. They were characterized by a positive charge promoting cell internalization and mucoadhesive behavior [12]. These results were in line with the relevance of surface modification of polymeric nanoparticles that has been evidenced in recent literature pointing out the positive effect of surface cationic charge on the interaction with biological substrates [13,14].

Some more work to elucidate the physico-chemical characteristics of these CS-OA-based nanosystems, and especially their interaction with cell substrates seems, however, still to be necessary.

This goal was afforded in the present paper by comparing CS-OA micelles and CS-OA PLGA NPs. Although both of the nanosystems compared are based on CS-OA, they present quite different structural characteristics. In the case of polymeric micelles, CS-OA represents the only component of the carrier, and the hydrophobic domains are represented by the self-assembled chains of oleic acid [7]. In the case of PLGA NPs, CS-OA is layered around the NPs, whose core is made by a matrix of the biodegradable polymer [12]. Therefore, different behavior could be envisaged for the two systems, concerning not only drug loading or release, but also interaction with cell substrates. A close comparison could guide in the future choice of the most suitable carrier according to the therapeutic objective.

The two carriers were loaded with resveratrol (RSV), a hydrophobic polyphenol from the stilbene family with interesting biological properties, strongly studied for its benefits in human health, such as anti-cancerogenic, anti-inflammatory, and heart/brain protective effects [15,16,17], but with low bioavailability due to poor aqueous solubility [18,19].

Most RSV benefits are, like for other polyphenols, strictly concentration dependent, so that at low concentrations, the antioxidant effect prevails, and RSV has a cancer preventive and anti-inflammatory protective effect, while at high concentrations pro-oxidant effect occurs, and ROS produced by RSV contribute to cancer cell growth arrest [20]. The concentration-dependent interaction of the RSV and RSV loaded NPs with cell substrates therefore seems especially useful to elucidate. In the present paper, the viability of two cell lines, Caco-2 cells, and Hela cells was studied following exposure to different concentrations of RSV in CS-OA-based micelles or NPs.

## 2. Results and Discussion

### 2.1. Particle Size and Zeta Potential

**Figure 1:**
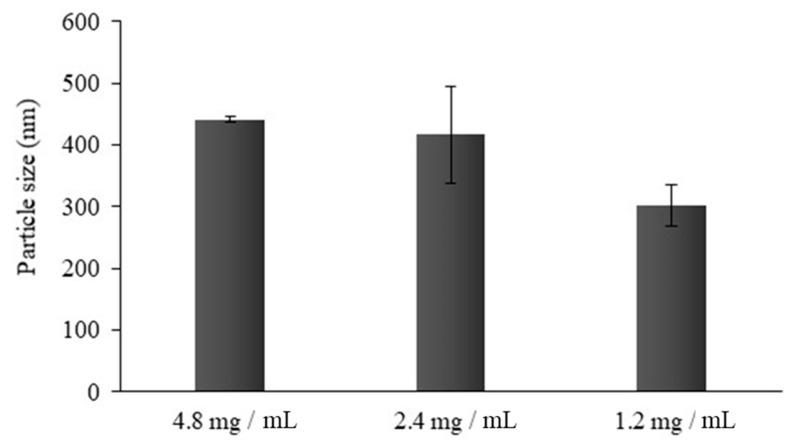
Average particle size (nm) of PLGA-NPs prepared with different CS-OA concentrations, corresponding to 1.5:1, 3:1 and 6:1 PLGA:CS-OA ratios (mean ± s.d.; n = 10).

The effect of CS concentration and of CS:oleic acid (OA) ratio on dimensions of CS-OA-based micelles was previously studied, indicating that higher concentration corresponded to larger dimensions, while no differences could be seen when 1:1 or 1:0.5 CS:OA molar ratio were compared [9]. For this reason, CS 0.05% (w/v) and a CS:OA molar ratio of 1:0.5 were used here. The relevance of CS-OA concentration on particle size was investigated for PLGA-NPs. The final concentration of CS-OA was varied among 4.8, 2.4, and 1.2 mg/mL, corresponding to a PLGA:CS-OA ratio in the final systems of 1.5:1, 3:1 and 6:1, respectively. Figure 1 shows the occurrence of a relevant and statistically significant decrease in particle size with a decrease in CS concentration from 4.8 to 1.2 mg/mL (one-way ANOVA, post-hoc Fisher’s test). This result does not seem to be in line with what had previously been observed [12], where a decrease in dimensions was seen when CS concentration was increased, an effect that was probably attributable to the distribution of CS-OA at the oil/water interface. It must be pointed out that in the present case, the range of CS concentration was higher than in the previous study. In the case of 4.8 and 2.4 mg/mL, the result can therefore be explained by the presence of excess CS-OA with respect to the amount necessary to saturate the droplet surface. As the amphiphilic character of the modified polymer determines its layering around the hydrophobic surface of the nanoemulsion droplets during NPs preparation, we can imagine that the exceeding CS-OA amount is not dispersed as well as the CS-OA that layers at the droplets’ surface, and a possible aggregation can occur.

On the basis of these data, a batch of PLGA-NPs was used in the present study, prepared with a CS-OA concentration of 1.2 mg/mL. The dimensions, the polydispersity index (PI), and the zeta potential of these NPs are reported in Table 1 for comparison with polymeric CS-OA micelles. The dimensional characterization of the RSV-loaded nanosystems is reported too. Both polymeric micelles and PLGA-NPs are characterized by a particle size in the nanometric range (250–300 nm), and a PI between 0.2 and 0.5, in line with results reported in previous work about similar systems [7,12], confirming the suitability of the preparation methods to obtain colloidal nanosystems with acceptable dispersion. The values of zeta potential are confirmed to be strongly positive for the unloaded nanosystems, in accordance with previous results [12], and RSV loading did not change these values.

### 2.2. Encapsulation Efficiency and Drug Loading

**Figure 2:**
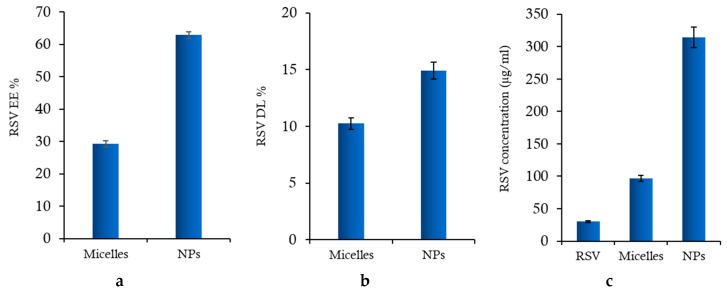
(**a**) Encapsulation efficiency (EE%), (**b**) drug loading (DL%) and (**c**) final RSV concentration, compared with RSV saturated solution (RSV), for polymeric micelles and PLGA NPs (mean values ± s.d.; n = 3).

Figure 2 shows the encapsulation efficiency (EE%), drug loading (DL%) values and final RSV concentration for both nanosystems. In NPs, RSV is entrapped in the inner lipophilic PLGA core during emulsification with CS-OA, while in polymeric micelles, it is positioned among the fatty acid chains during the occurrence of self-assembling micelle hydrophobic domains. As illustrated, RSV is efficiently loaded in polymeric NPs with an EE% value two-fold higher than in micelles (Figure 2a). The DL% values (Figure 2b) are in line with those of EE and support the different distribution of RSV into the two types of nanosystems. Figure 2c illustrates the final concentrations of RSV in colloidal dispersions of micelles and PLGA-NPs compared to RSV saturated solution. The increase in concentration is about three times in the case of polymeric micelles, and about ten times in the case of NPs, suggesting the potential positive effect of both nanosystems in overcoming the bioavailability problems of RSV due to its low solubility. The results obtained evidence the higher efficiency of NPs as a carrier with respect to polymeric micelles, probably due to the presence of the PLGA core, in which more RSV molecules can be loaded with respect to the hydrophobic domains occurring by self-assembling of oleic acid chains inside the CS-OA polymeric micelles.

### 2.3. Physico-Chemical Characterization

#### 2.3.1. Thermal Analysis Characterization

**Figure 3:**
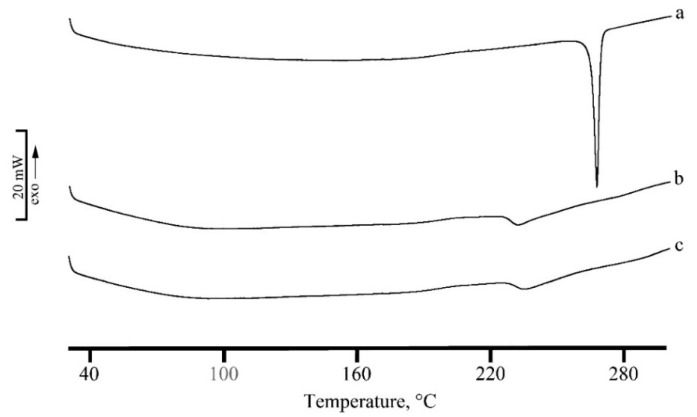
DSC profiles of RSV (**a**), unloaded micelles (**b**), and RSV loaded micelles (**c**). The y bar scale corresponds to 20 mW.

**Figure 4:**
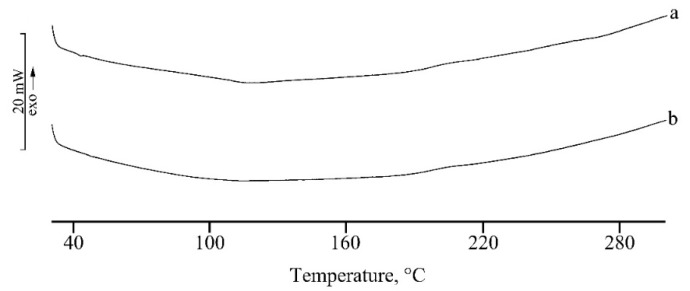
DSC profiles of unloaded PLGA-NPs (**a**), and RSV loaded PLGA-NPs (**b**). The y bar scale corresponds to 20 mW.

The results of DSC analysis performed on both the nanosystems, unloaded and RSV loaded, are given in Figure 3 and Figure 4 for micelles and PLGA-NPs, respectively.

RSV is a commercial microcrystalline white powder with a thermal profile typical of an anhydrous drug (Figure 3a) with a melting endothermic effect at T_peak,m_ = 265.1 °C (T_onset,m_ = 263.1 °C; ΔH_m_ = 281 J g^-1^). This peak disappeared in the DSC profile of RSV loaded micelles where only an exo-endothermic effect due to CS-OA decomposition is recorded (Figure 3 curve c). This thermal profile suggests the encapsulation of RSV in the micellar structure.

A similar thermal behavior is recorded for PLGA-NPs, where the entrapment of RSV in NPs and its homogeneous dispersion in the polymeric matrix is confirmed by the disappearance of its melting effect in DSC curve of RSV loaded NPs (Figure 4b).

**Figure 5:**
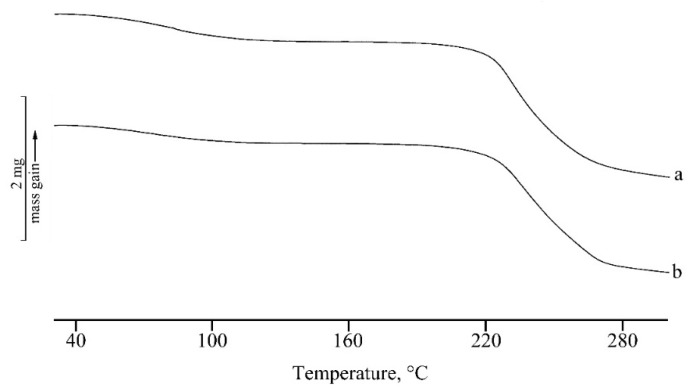
TGA curves of unloaded (**a**) and RSV-loaded (**b**) micelles. The y bar scale corresponds to 2 mg of weight variation.

**Figure 6:**
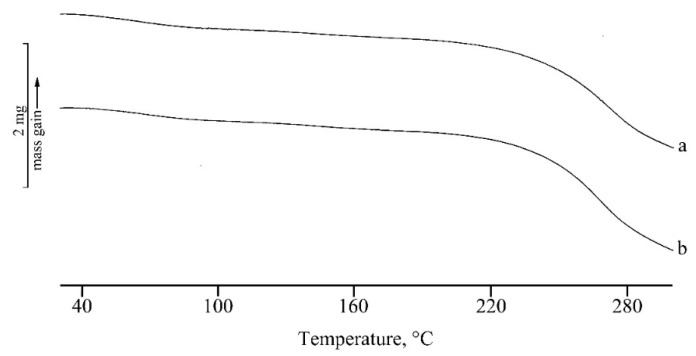
TGA curves of unloaded (**a**) and RSV-loaded (**b**) PLGA-NPs. The y bar scale corresponds to 2 mg of weight variation.

In Figure 5 and Figure 6, the TGA curves of the unloaded (curves a) and loaded (curves b) nanosystems are reported for micelles and NPs, respectively. The first mass loss (about 7%) is due to the dehydration of the lyophilized samples and the second one to decomposition. The presence of RSV does not influence the thermal stability for NP systems that show exactly the same temperatures of onset (T_onset, dec_ = 189.2 ± 0.3 °C) and very close temperatures of peak (maximum rate of weight loss), as obtained from the calculation of the first derivative (about 279 °C for both the systems). In the case of the micelles, the same temperature of peak was observed for unloaded and loaded samples (T_onset, dec_ = 165.8 ± 0.2 °C). A small difference in decomposition peak temperatures can be evidenced in this case between 237 ± 2 °C for the unloaded sample and 243 ± 3 °C for the RSV loaded one, suggesting quite a small stabilization effect due to the presence of the drug. As the self-assembling of the NPs is driven by the hydrophobic interactions between fatty acid moieties, it seems conceivable that the presence of the hydrophobic molecule of RSV plays a stabilization effect. This mechanism could be even more relevant in the case of PLGA-NPs, in which the fatty acid chains interact during the preparation, with the hydrophobic PLGA core of the NPs. These in fact appear to be more stable than micelles, with higher values of thermal stability (189 °C versus 166 °C for T_onset,dec_ and 279 °C versus about 237–243 °C for T_peak,dec_).

#### 2.3.2. ATR Fourier-Transform Infrared (FT-IR) Spectroscopy

**Figure 7:**
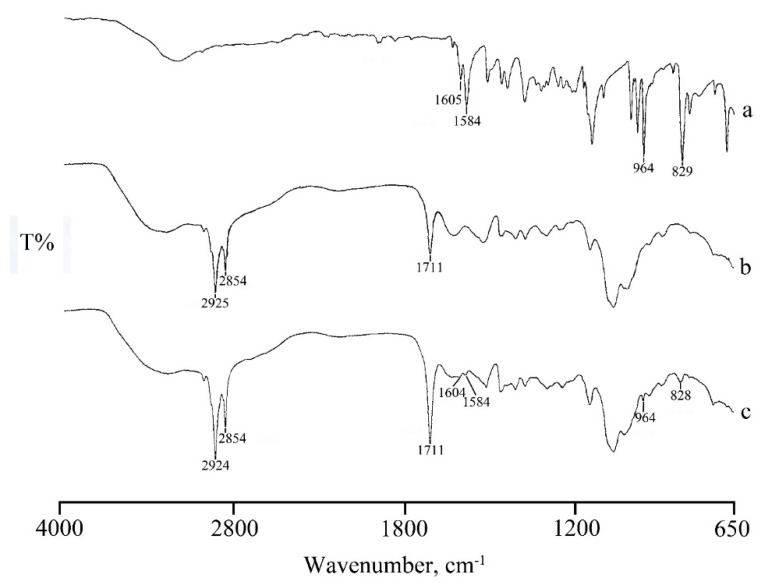
FTIR spectra of RSV (**a**), and unloaded (**b**) and RSV-loaded (**c**) micelles.

The FT-IR spectrum of RSV (Figure 7a) presents a broad band at around 3200 cm^−1^ due to the O-H stretching vibration, a set of typical bands at 1605, 1584 cm^−1^ corresponding to C-C aromatic double bond stretching and C-C olefinic stretching, respectively, a band at 964 cm^−1^ attributable to trans olefinic bond, and a band at 829 cm^−1^ due to the bending vibration band of C-H in aromatic rings [21].

**Figure 8:**
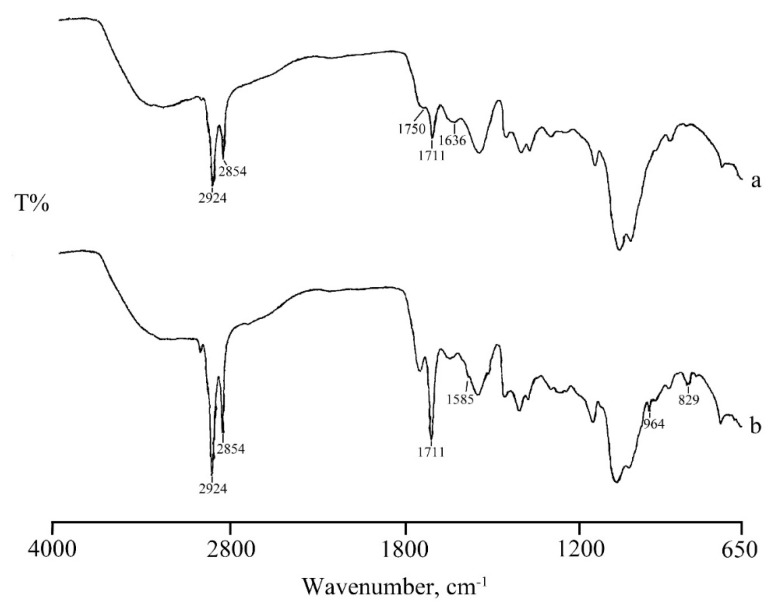
FTIR spectra of PLGA-NPs: unloaded (**a**) and RSV-loaded (**b**).

In the FT-IR spectrum of RSV loaded micelles (Figure 7c) in addition to the characteristic band of CS-OA C=O stretching at 1711 cm^−1^, the presence of the typical RSV bands suggest that RSV was encapsulated in the core of the micelles through hydrophobic interactions, supporting the thermal data. Also, in NP systems the loading of RSV is confirmed by the FT-IR spectra (Figure 8). The spectrum of NPs (Figure 8 spectrum a) shows bands at 1750 cm^−1^ and 1711 cm^−1^ due to stretching vibration of C=O in PLGA and in CS-OA, respectively, and a band at 1636 cm^-1^ due to C-O-O stretching vibration of PLGA. The same bands appear also in the RSV-NPs sample (Figure 8b) where the characteristic bands of RSV loaded in the polymeric material are also evident.

#### 2.3.3. X-ray Diffraction Characterization

**Figure 9:**
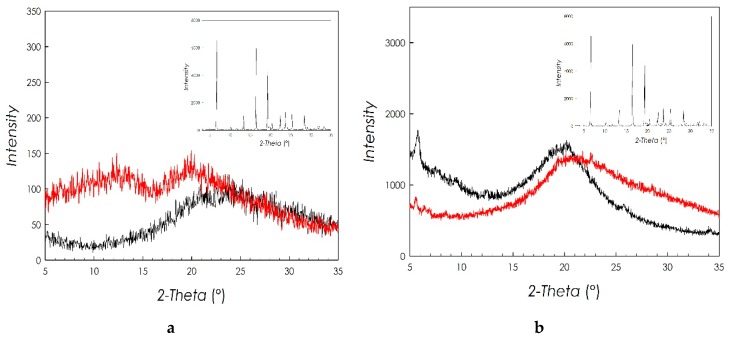
(**a**) XRD pattern of unloaded micelles (black curve) and RSV-loaded micelles (red curve); (**b**) XRD pattern of unloaded NPs (black curve) and RSV-loaded NPs (red curve); in both Figures the XRD pattern of RSV is reported in the inset.

The XRD patterns of micelles and PLGA-NPs are reported in Figure 9a,b, respectively. In both Figures, the black curve refers to unloaded nanosystems, the red one to nanosystems loaded with RSV, whose diffraction pattern is reported in the inset.

It is possible to see that both unloaded systems present a typical diffraction pattern, pertaining to an amorphous system. This characteristic is also found in the samples loaded with RSV.

On the other hand, the diffraction pattern of the RSV shows the typical features of a good crystalline material with well-defined and narrow diffraction peaks. The absence of any diffraction in the patterns of RSV loaded micelles and NPs allows to conclude that RSV becomes amorphous during the preparation of both nanosytems, confirming the results of the DSC analysis. This result suggests that RSV is dispersed at the molecular level both inside the hydrophobic domains of the polymeric micelles and inside the PLGA polymeric matrix, which represents the core of CS-OA-coated PLGA NPs. It can be argued that this is due to the good affinity of the drug for the fatty acid and for the PLGA chains inside the two nanocarriers.

### 2.4. Drug Release Profiles

**Figure 10:**
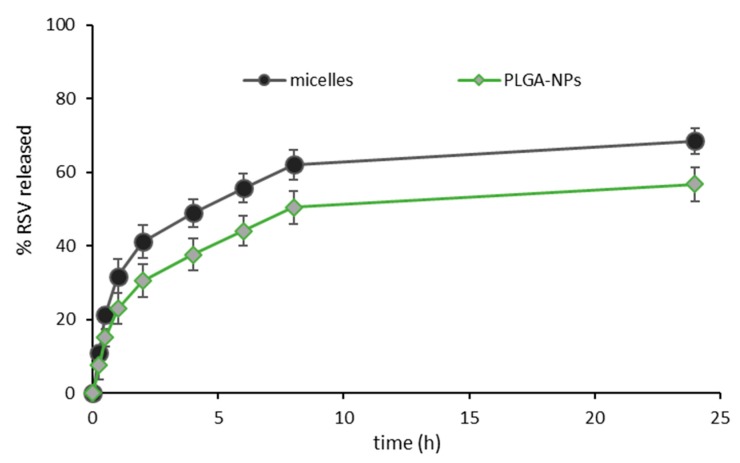
RSV release profiles from micelles and PLGA-NPs (mean values ± s.d.; n = 3).

In Figure 10, the release profiles of RSV from the two types of nanosystems are reported. In both cases, a fast initial release was followed by a slower phase. This seemed conceivable for PLGA NPs, that require polymer degradation to complete the RSV release from the inner core [22]. The degradation depends on several factors, encompassing polymer characteristics, but also the presence of the drug and of other components, the particle dimensions and the environmental conditions, and it is likely to require some days to be completed [23]. For the micelle system, a slightly higher release profile was observed, as at each time point, a greater % of RSV was released from micelles than from PLGA-NPs. However, the difference did never exceed 12%, indicating that the structure of micelles was more tough than it could be expected considering that it is based on weak electrostatic and hydrophobic interactions. It can be argued that the high number of interactions involved along a single chitosan chain can anyway stabilize the NPs, despite their weak nature, and of the potential sensitivity to the presence of salts in the release medium.

### 2.5. Biocompatibility

**Figure 11:**
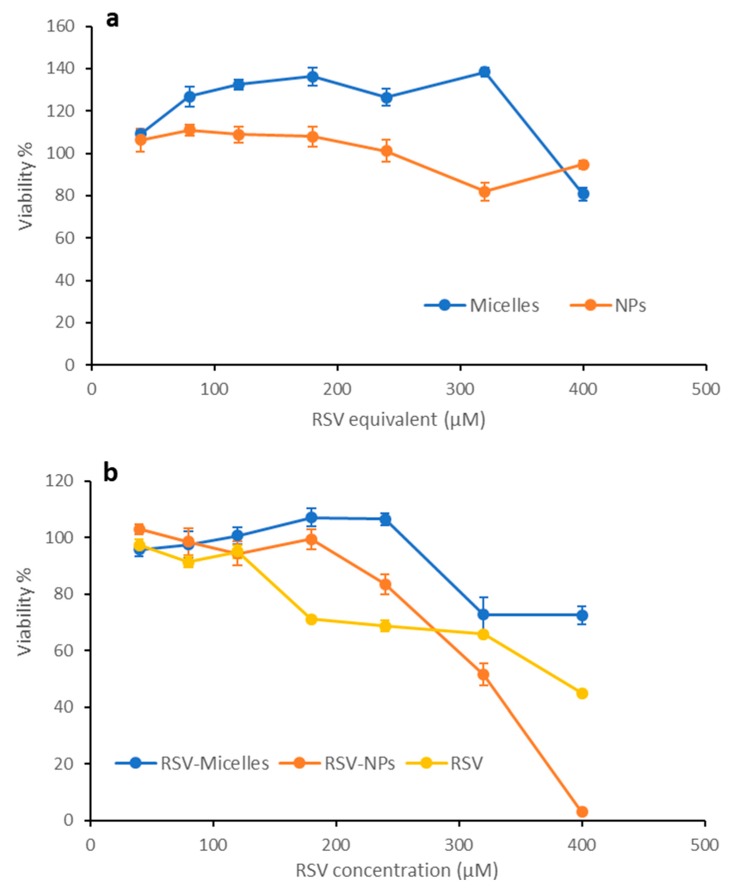
Viability of CaCo-2 cells treated with free RSV or nanosystems based on CS-OA, as a function of RSV concentration. Unloaded nanosystems were tested at the same concentrations of the loaded ones (indicated as RSV equivalent concentration). (**a**) Unloaded nanosystems; (**b**) free RSV and RSV-loaded nanosystems (mean values ± s.d.; n = 8).

**Figure 12:**
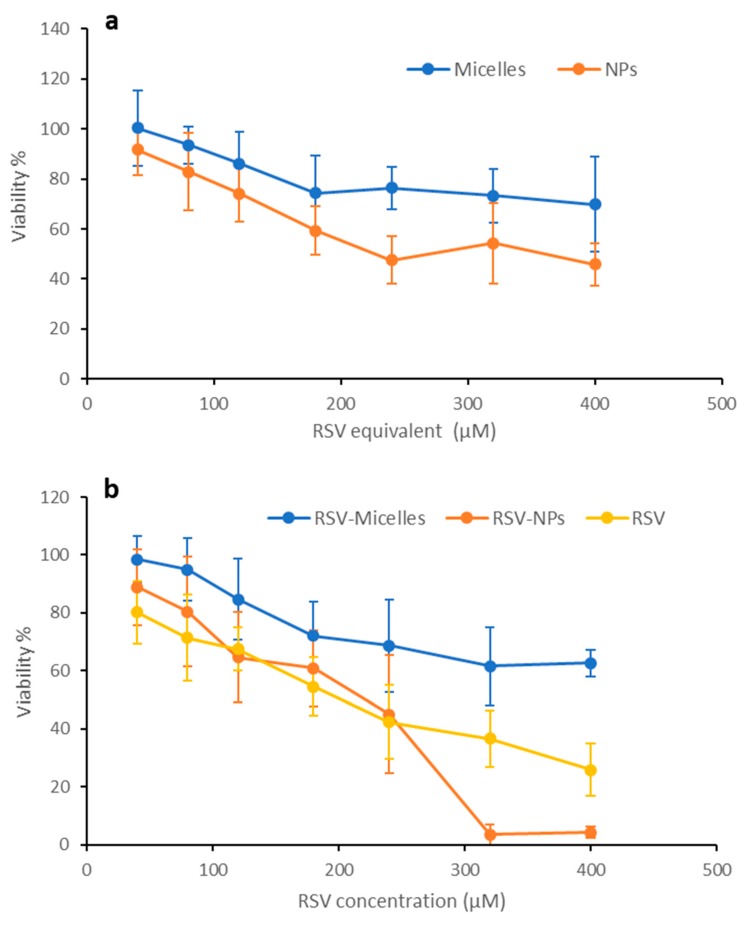
Viability of HeLa cells treated with free RSV or nanosystems based on CS-OA as a function of RSV concentration. Unloaded nanosystems were tested at the same concentrations of the loaded ones (indicated as RSV equivalent concentration). (**a**) Unloaded nanosystems; (**b**) free RSV and RSV-loaded nanosystems (mean values ± s.d.; n = 8).

The results of the MTT test carried out on the two cell lines, Caco-2 and HeLa, are shown in Figure 11 and Figure 12, respectively. The percentage of viability after treatment with the two loaded nanosystems (Figure 11b and Figure 12b) is compared with the values measured after contact with free RSV, tested at same concentrations. Unloaded systems were assessed at concentrations comparable to those used for RSV loaded ones (Figure 11a and Figure 12a).

Looking at the unloaded systems, no considerable cytotoxic effects took place when Caco-2 cells were treated with both nanosystems, that resulted compatible with good cell vitality (more than 80%) until the highest concentration (Figure 11a). HeLa cells (Figure 12a) resulted more sensitive to the exposure to unloaded micelles, with a viability of about 70% at the highest concentration, and especially to the exposure to PLGA-NPs, that induced a residual viability of about 45% at the highest concentration tested.

These same differences between the two cell lines can be seen in the case of free RSV and of RSV loaded nanosystems (Figure 11b and Figure 12b).

Concerning free RSV, the results here obtained are in line with those reported in the literature [24,25].

In contrast, when the Caco-2 cell line was exposed to RSV-loaded nanosystems, a more evident dose-dependent cytotoxic effect is revealed; in particular, in the case of PLGA-NPs, a higher and significant reduction in viability in comparison with free RSV and polymeric micelles occurs (Figure 11b). While viability for micelles remains above that of free RSV, in the case of PLGA-NPs at the highest concentrations (300 and 400 µM) the viability is lower than for free RSV. These results are in line with similar data obtained with other colon cancer cell lines such as HT29 and LS147T [25].

HeLa cells appear more sensitive than Caco-2 cells to the treatment with unloaded nanosystems, with viability levels lower than in the case of Caco-2 cell line. In the case of RSV loaded nanosystems, however, a quite similar trend can be observed for the two cell lines. For PLGA-NPs, viability decreases more quickly with concentration increase than in the case of free RSV and of RSV loaded micelles. As shown in Figure 12b, for polymeric micelles cell viability remained higher than for RSV at all concentrations. With a trend quite similar to that observed in Caco-2 cells, PLGA-NPs at concentrations higher than 250 µM seems to determine higher cytotoxicity with respect to free RSV. This difference suggests that the different structure of nanosystems can markedly affect their interaction with the cell substrate, conceivably due to different amount of drug internalization. In this process, a role is conceivably played by CS-OA, which thanks to its hydrophilic/hydrophobic balance and to the presence of positive surface charges, makes the colloidal systems suitable for stronger interaction with the cell membranes, and more efficiently subject to endocytosis [26].

### 2.6. Cell Internalization Properties

**Figure 13:**
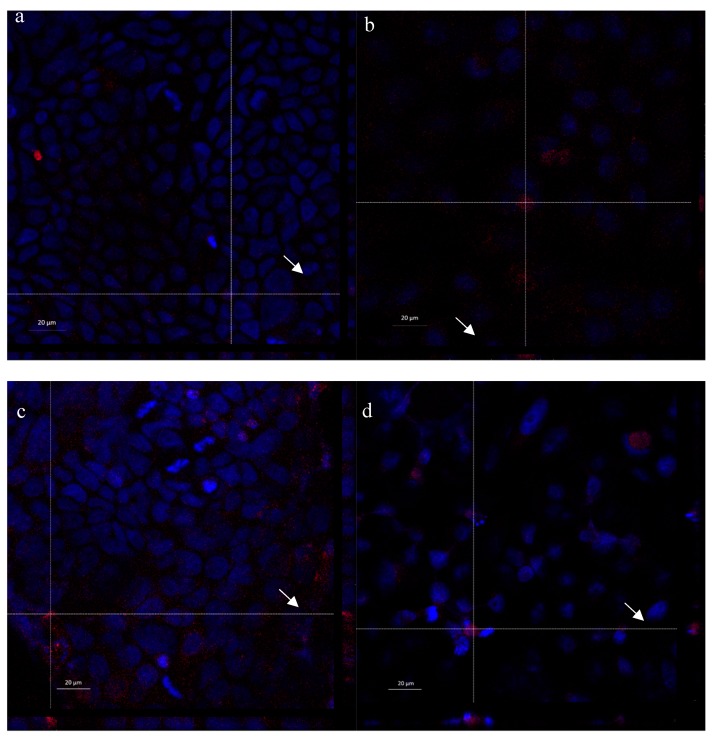
CLSM of Caco-2 and HeLa cells treated with the two nanosystems loaded with Nile red. (**a**) Micelles on Caco-2 cells, (**b**) micelles on Hela cells, (**c**) PLGA-NPs on Caco-2 cells, (**d**) PLGA-NPs on HeLa cells.

**Figure 14:**
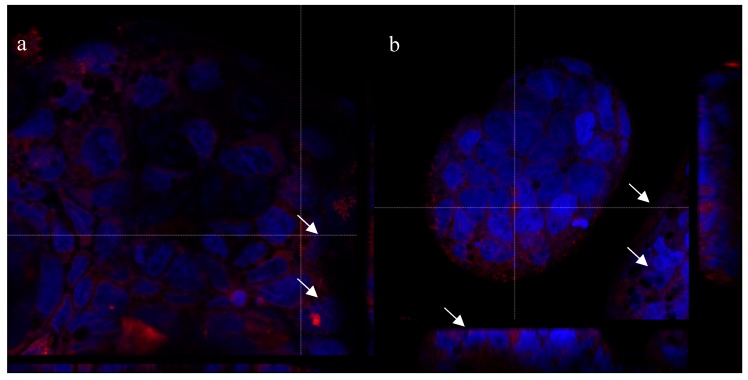
CLSM of Caco-2 cells treated with nanosystems prepared with CS-OA rhodamine labeled. (**a**) Micelles, (**b**) PLGA-NPs.

In Figure 13, Laser Scanning Confocal Microscopy (CLSM) photographs of Caco-2 and HeLa cell lines treated with polymeric micelles and PLGA-NPs loaded with Nile red are reported. Confocal images confirm the presence of Nile red fluorescence signal inside the cells in all the samples; in particular, the red dye appears not only around, but also close to the blue-stained nuclei, as it is possible to appreciate by looking at the Z-axis projections (arrows). The localization of red staining in well-defined spots suggests the internalization of the nanosystems instead of the Nile red released from them. However, to better investigate this aspect, both micelles and PLGA-NPs were prepared using as stabilizer rhodamine labeled CS-OA [27]. In this case, the red staining indicates the presence of the labeled polymer. CLSM images of Caco-2 cells treated with these nanosystems (Figure 14) show the internalization of rhodamine, with a localization in the cytosol and close to the nuclei, similarly to what was previously observed for Nile red-loaded nanosystems. This result suggests that both nanocarriers are effectively included in the cells. It is conceivable that the nanometric particle size of both nanosystems and the ability of interaction of positively charged chitosan shell with cell membranes played a role in triggering an endocytic mechanism.

CLSM visualization does not allow a clear differentiation between the samples treated with PLGA-NPs and micelles, in line with the structure of micelles more tough than expected, as evidenced by the RSV release test. However, the biocompatibility test suggests a different behavior for the two nanosystems, which can be explained by the hypothesis that the micelles have a less compact structure and lower stability with respect to PLGA-NPs, which is suggested also by thermal analysis.

## 3. Materials and Methods

### 3.1. Materials

Chitosan LMW (CS) (80% Deacetylation Degree, DD), Poly lactic-glycolic acid (PLGA) (Resomer RG 503H), Nile red were purchased from Sigma-Aldrich (Milan, Italy). Oleic acid (OA) was purchased from Fluka (Milan, Italy) and 99% Pure Trans-Resveratrol (RSV) from Mega Resveratrol (Candlewood Stars Inc., Danbury, CT, U.S.A.). Acetone, ethyl acetate, acetic acid, sodium acetate, and methanol were acquired from Carlo Erba (Milan, Italy).

### 3.2. Methods

#### 3.2.1. Preparation of the Nanosystems

As reported in previous works [7,9], polymeric micelles were obtained starting from a 0.5 mg/mL chitosan HCl solution. HCl salt was in turn obtained from low molecular weight (LMW) chitosan base, deacetylation degree (DD) 80% (Sigma-Aldrich, Milan, Italy), by addition of HCl 0.5 N to chitosan until complete dissolution, dialysis in bidistilled water for 24 h and freeze-drying (HetoDrywinner, Analitica de Mori, Milan, Italy). Considering the 80% DD, the 1:0.5 CS:OA molar ratio corresponded to 0.7 mg of oleic acid per each mg of chitosan. OA was dissolved in acetone and dropwise added to the aqueous solution of CS under stirring. After solvent evaporation under stirring overnight, a micelle dispersion was obtained, that was sonicated (Elmasonic S 80 H, Elma Hans Schmidbauer GmbH & Co, Singen, Germany) in an ice bath for 15 min. To obtain loaded micelles, RSV was dissolved in acetone together with OA, at a final concentration of 0.33 mg/mL. To remove RSV not encapsulated the samples were centrifuged (ALC 4218 centrifuge) for 10 min at 3000 rpm. RSV loaded micelles were prepared in amber glass vials.

To prepare PLGA-NPs, CS was added to 100 mL of bidistilled water under magnetic stirring (300 rpm) containing 250 µL of glacial acetic acid to obtain a 1% w/w polymer concentration. To functionalize the 50% of CS binding sites with OA, a stoichiometric amount of fatty acid was solubilized in acetone and added dropwise to the CS solution, to obtain chitosan oleate (CS-OA). After acetone evaporation, CS-OA was freeze dried.

Freeze-dried CS-OA was employed to obtain PLGA-NPs by partially modifying the solvent evaporation method previously described [12]. CS-OA was dispersed in 3 mL of distilled water and 0.25 mL of ethyl acetate solution containing 24 mg/mL of PLGA were added during the emulsification step. This step was carried out at 20,500 rpm by means of Ultra-Turrax T25 (Janke & Kunkel, IKA^®^ Labortechnik, Germany) equipped with 8 mm probe (S25 N-8 G). After 5 min, 7 mL of distilled water was added, and emulsification was carried out for further 5 min. Then, ethyl acetate was removed under stirring overnight. The weight lost due to evaporation was determined and the initial volume (10 mL) was reconstituted with distilled water. Finally, NPs were sonicated for 15 min in an ice bath and centrifuged for 10 min at 3000 rpm. Different CS-OA concentrations were employed (4.8, 2.4 and 1.2 mg/mL). To obtain RSV-loaded PLGA-NPs, RSV was added to the organic phase together with PLGA, in a final concentration of 0.5 mg/mL. RSV-loaded NPs were prepared in amber glass vials.

Rhodamine-labeled CS was obtained according to the literature [27]. Briefly, 200 mg of CS was dissolved in 20 mL of acetic acid overnight. 20 mL of anhydrous methanol was added to the solution under magnetic stirring for 3 h, and nitrogen was flushed for 15 min to remove the oxygen present. 13.8 mg of rhodamine B isothiocyanate (RITC) (Sigma Aldrich, Milan, Italy) was solubilized in anhydrous methanol at a concentration of 2 mg/mL and added dropwise to the CS solution under constant stirring. The reaction was kept at room temperature and in the dark for 18 h. CS-RITC was precipitated using 10 N NaOH. Dialysis cycles in distilled water, in HCl 0.5 N, and in distilled water again were performed before freeze drying.

#### 3.2.2. Particle Size and Zeta Potential

The mean particle size and the polydispersity index (PI) of the samples were assessed by means of Photon Correlation Spectroscopy (PCS; N5 submicron particle size analyzer, Beckman Coulter, Milan, Italy). Analyses were carried out at a 90° detection angle, at room temperature, by diluting 50 μL of the sample in 3 mL of distilled and filtered (0.22 µm) water. PI is a measure of particle size distribution usually ranging between 0.05 for highly monodisperse samples and 0.7 for broadly dispersed ones.

Zeta potential was evaluated by a Zetasizer^®^ nano series (Malvern Instruments Ltd., Worcestershire, United Kingdom) in aqueous suspension, with conductivity ranging between 0.263 and 0.479 mS/cm, by setting a dielectric constant of 78.5 and a refractive index of 1.33.

#### 3.2.3. Encapsulation Efficiency and Drug Loading

The % RSV amount encapsulated in the nanosystems was calculated by difference according to Equation (1). The nanosystems were centrifuged at 3000 rpm for 10 min. The free drug precipitated was solubilized in mobile phase and analyzed by HPLC (Perkin-Elmer Series 200 HPLC) equipped with a C18 column (125 Angstrom, Waters, 150-mm length, 4.6-mm inner diameter, packing size 10 μm) and using as mobile phase methanol:water:acetic acid 52:48:0.05 (*v*/*v*). The analysis was performed at 25 °C in isocratic conditions (flow rate 1 mL/min), using UV detection at 303 nm wavelength. Injection volume was 20 μL.

% encapsulation efficiency was measured according to the following Equation (1):

(1) $$\%EE=\frac{Wi-Wf}{Wi}*100$$

where Wi = total drug amount; Wf = free drug amount.

Based on the results of EE%, the percentage drug loading (%DL) was calculated using the following Equation (2):

(2) $$\%DL=\frac{We}{We+Wexcipients}*100$$

where We = encapsulated drug amount, derived from EE%; Wexcipients = excipients amount in the nanosystems.

RSV concentration in final colloidal solution was calculated by difference between the initial RSV concentration and the RSV amount not encapsulated and removed by centrifugation.

### 3.3. Physico-Chemical Characterization

#### 3.3.1. Differential Scanning Calorimetry (DSC)

DSC analyses were performed with a Mettler STAR^e^ system (Mettler Toledo, Novate Milanese, MI, Italy) equipped with a DSC821^e^ Module and an Intracooler device for sub-ambient temperature analysis (Julabo FT 900) on 2–3 mg (Mettler M3 Microbalance) samples in sealed aluminum pans with pierced lid (heating rate β = 10 K min^−1^, nitrogen air atmosphere (flux 50 mL min^−1^), 30–300 °C temperature range)). The instrument was previously calibrated with Indium as standard reference. Measurements were carried out at least in triplicate.

#### 3.3.2. Simultaneous Thermogravimetric Analysis (TGA/DSC)

Mass losses were recorded with a Mettler STAR^e^ system (Mettler Toledo, Novate Milanese, MI, Italy) TGA with simultaneous DSC (TGA/DSC1) on 3–4 mg samples in alumina crucibles with lid (heating rate β = 10 K min^−1^, nitrogen air atmosphere (flux 50 mL min^−1^), 30–300 °C temperature range). The instrument was previously calibrated with Indium as standard reference and measurements were carried out at least in triplicate.

#### 3.3.3. ATR Fourier-Transform Infrared (FT-IR) Spectroscopy

IR spectra were recorded using a Fourier transform infrared spectrophotometer (Perkin Elmer SpectrumOne, Monza, Italy) with a single reflection ATR accessory (PIKE MIRacle™). Approximately 10mg of each sample were placed on ATR crystal of ZnSe and pressed down to the crystal. The spectra were collected with a resolution of 4 cm^−1^ within the spectral range of 650–4000cm^−1^.

#### 3.3.4. X-ray Analysis

X-ray diffraction data were collected on micelles and PLGA-NPs, both unloaded and loaded with RSV, by means of a D8 Bruker Advance diffractometer equipped with a Cu source. Samples have been measured in a zero-background sample holder in the 5–35° range, step/size 0.04°, counting time 10 s/step.

### 3.4. Release Test

A dialysis bag method was employed to assess drug release from the nanosystems. Briefly, the dialysis bag (cellulose acetate, cutoff 12–14,000 Da) was filled with 5 mL of the NP suspension (diluted to obtain a RSV amount compatible with the release in sink conditions) and was placed in 45 mL of Phosphate Buffered Saline (PBS)/EtOH (90:10 *v*/*v*) as receiving phase. Samples (45 mL) of receiving phase were withdrawn at fixed times, ranging from 15 min to 24 h. The sink conditions were ensured by replacing the volume withdrawn at each sampling time with fresh receiving phase. The drug amount was quantified by the HPLC method previously described.

### 3.5. Cytotoxicity Assay

The cytotoxicity of micelles and PLGA-NPs (unloaded and loaded with RSV) and free RSV suspended in PSB/EtOH (90:10 *v*/*v*) was assessed against two different human cancer cell lines: colonic adenocarcinoma (Caco-2) and human cervical cancer (HeLa) cell lines. Cells were sub-cultured in Dulbecco’s Modified Eagle Medium (DMEM) supplemented with 1% *v*/*v* antibiotic/antimycotic solution and 10% *v*/*v* inactivated fetal calf bovine serum; 1% *v*/*v* of non-essential amino acid was added to Caco-2 medium. Both cell types were seeded in 96-well plates (2.5 × 10^4^ cells in 200 μL medium/well) and incubated (37 °C and 5% CO_2_ atmosphere) for 24 h to reach semi-confluence. All samples were diluted with medium to obtain different RSV concentrations (40, 80, 120, 180, 240, 360, 400 μM) calculated on the basis of EE% results. The same dilutions were performed for the unloaded carriers to obtain concentrations comparable to those of the loaded ones. Cells were treated with 200 μL of each sample solution for 24 h and medium was used as reference. After 24 h contact, an MTT assay was performed. Cells were washed with 100 µl of PBS (pH 7.4) and then incubated for 3 h (37 °C and 5% CO_2_) with 50 μL of an MTT 7.5 μM solution in 100 μL of DMEM, without phenol red. Finally, 100 μL of dimethylsulfoxide (DMSO) was added to each well, to allow the complete dissolution of formazan crystals, obtained from MTT dye reduction by mitochondrial dehydrogenases of cells alive. The solution absorbance was determined, after 60 s shaking, at 570 nm, with a 690 nm reference wavelength, by means of an IMark1 Microplate reader (Bio-Rad Laboratories S.r.l., Segrate, Milan, Italy). Results were expressed as % viability calculated by normalizing the absorbance measured after contact with samples with the absorbance of the positive control (cells alive in medium). Eight replicates were performed for each sample.

#### Laser Scanning Confocal Microscopy (CLSM)

CLSM analyses were performed on samples labeled with two different fluorescent tracers. Micelles and PLGA-NPs were loaded with Nile red to track the drug fate. At the same time, cell internalization of micelles and NPs was assessed by preparing nanosystems with a modified rhodamine labeled CS-OA [27]. The CLSM analyses were conducted as described below: a microscope slide (Ǿ = 13 mm) was inserted into each well in a 24-well plate, and 10 × 10^5^ cells were seeded in 500 μL medium. After 24 h culture, samples, diluted with medium to reach a 20 μM drug concentration, were loaded onto the cells and left in contact for further 24 h. Afterwards, cells were washed twice with 500 µL of PBS and fixed for 15 min at 4 °C with a 4% (*v*/*v*) paraformaldehyde solution diluted in PBS. Then, substrates were flushed with 500 µL of PBS and, before confocal microscopic analysis, were treated in the dark with 100 µL of Hoechst 33258 (diluted 1:10000 in PBS) for 15 min. Finally, Hoechst was removed, and cells were washed with PBS. The slides with fixed cells were removed from the wells and were examined with confocal laser scanning microscopy (CLSM, Leica TCS SP5II, GmbH) by setting the fluorescence of labeled nuclei (Hoechst 33258, λ_ex_ = 346 nm and λ_em_ = 460 nm), Nile red (Nile red, λ _ex_ = 440 nm and λ_em_ = 520 nm) and rhodamine CS-OA (λ_ex_ = 553 nm; λ_em_ = 627 nm).

### 3.6. Statistical Analysis

Statistical evaluations were performed by means of Statgraphics 5.0, Statistical Graphics Corporation, MD, USA. Differences were determined according to one-way ANOVA, post-hoc Fisher’s test and were considered significant at *p* < 0.05.

## 4. Conclusions

The results presented in this paper confirm that CS-OA, for its ability to self-assemble and to stabilize emulsions, is suitable for preparing both micelles and PLGA-NPs containing RSV as lipophilic model drug. The two types of nanocarriers were characterized by particle size lower than 300 nm and drug loading capacity between 10 and 15%, confirming the affinity of the RSV molecule for the hydrophobic core of the nanosystems. The effectiveness of CS coating was verified by positive values of zeta potential. Even though physical properties of polymeric micelles and PLGA core NPs looked similar, the interaction of the two systems loaded with RSV onto Caco-2 and Hela cells was different. Only PLGA core-loaded NPs determined a strong decrease in cell viability at high concentrations, in comparison with loaded micelles and free RSV, with a Lethal Dose 50% (LD50) value measured at 240 μM. This result can be attributed to the different hydrophobicity of the two types of nanocarriers and to the more compact nanostructure of PLGA core. The CS-OA micelle structure appeared more stable than expected, but probably less stable than that of PLGA-NPs. CLSM microphotographs confirmed the good internalization of the entire nanocarriers into the cytosolic portion of the cells. Thanks to their lipophilicity, both polymeric carriers based on CS-OA are promising candidates to enable molecular dispersion of the loaded drug, characterized by suitable drug loading, size and cell internalization properties. So far, their use for topical drug delivery systems is envisaged: in particular, depending on the dose of RSV, two different goals can be targeted. At low RSV concentration, nanocarriers could be useful for skin wound treatment, thanks to the peculiar antioxidant and anti-inflammatory properties of the drug that can accelerate the wound healing process. At high RSV concentrations, a cytotoxic effect is expected from PLGA-NPs, which could be profitably exploited for the topical treatment of skin tumors.

## Figures and Tables

**Table 1:** Comparison between the dimensions of the two nanosystems, both unloaded and loaded with RSV (mean ± s.d.; n = 3).

		Mean Diameter (nm) ± sd	PI ± sd	Zeta Potential (mV) ± sd
**unloaded**	Micelles	266 ± 1	0.51 ± 0.08	54.1 ± 1.2
	PLGA-NPs	30 ± 4	0.53 ± 0.03	53.6 ± 0.8
**RSV loaded**	Micelles	289 ± 13	0.33 ± 0.06	57.9 ± 1.1
	PLGA-NPs	273 ± 3	0.24 ± 0.02	54.6 ± 1.2
